# A Comparison of Two Different Methods for Inducing Apnoea During Thoracic Computed Tomography of Dogs

**DOI:** 10.3390/ani15071014

**Published:** 2025-04-01

**Authors:** Thomas Hordle, Maria Navarro-Carrillo, Imogen Schofield, Mark Plested, Maria Chie Niimura del Barrio

**Affiliations:** Lumbry Park Veterinary Specialists (CVS), Alton GU34 3HL, UK; maria.carrillo@cvsvets.com (M.N.-C.); mark.plested@cvsvets.com (M.P.)

**Keywords:** apnoea, CT, IPPV, midazolam, atelectasis, dog, anaesthesia

## Abstract

Apnoea may be induced by pharmacological or ventilatory measures in animals under general anaesthesia to prevent motion blur on thoracic computed tomography (CT) sequences. The effectiveness of such methods has previously been reported in terms of CT image quality but not in the success of apnoea induction. Therefore, the purpose of this study was to evaluate the efficacy of two different methods used to induce apnoea. A total of 61 client-owned dogs undergoing general anaesthesia for thoracic CT were randomised to group M (*n* = 30) and group V (*n* = 31). In group M, midazolam 0.2 mg kg^−1^ was administered by rapid intravenous injection. In group V, dogs were mechanically ventilated to maintain normocapnia, before pausing the ventilator for image acquisition. Apnoea was induced in 77% of dogs in group M and 94% in group V (*p* = 0.08). The onset of apnoea was quicker and of a longer duration in group V (*p* < 0.001). Changes in cardiorespiratory parameters and the degree of atelectasis were greater following midazolam administration. For these reasons, the authors recommend interruption of mechanical ventilation to induce apnoea in suitable dogs, as it enables the period of apnoea to be more controlled and repeated if required.

## Simple Summary

To prevent movement blur, patients must remain still and not breathe for computed tomography (CT), an advanced medical imaging modality of the chest. Whilst people can be instructed to do this, animals must be anaesthetised and given a drug or their lungs ventilated to temporarily stop their breathing, so-called apnoea. This study’s main aim was to compare two methods used commonly in the authors’ institution to induce apnoea in dogs. One was the administration of midazolam, a drug that depresses breathing among multiple other applications. The other was using a ventilator to mechanically deliver breaths, overcoming the patient’s own drive to breathe, before pausing the machine for the chest CT scan. Both methods had a similar efficacy in over three quarters of the dogs investigated but the use of the ventilator resulted in improved image quality and a greater degree of control, with fewer side effects. The onset and duration of apnoea induced by each method was also described for the first time, being quicker and longer, respectively, when using the ventilator compared to midazolam. These findings will enhance the understanding of veterinary professionals on how to safely and effectively induce apnoea in dogs under general anaesthesia.

## 1. Introduction

Computed tomography (CT) is typically performed under general anaesthesia or sedation in animals so that they do not move during image acquisition [1,2]. Various anaesthetic techniques that induce apnoea or breath-holding may be utilised to prevent respiratory motion artefacts during thoracic imaging too. By contrast, conscious human patients may simply be requested to remain still and hold a full inspiration during thoracic CT imaging [2,3].

Apnoea is the temporary cessation of spontaneous breathing, which may be induced by pharmacological respiratory depression or interruption of intermittent positive pressure ventilation (IPPV) [1,2,3,4], whereas a breath-hold is achieved by ventilatory measures that maintain a certain positive inspiratory pressure (PIP) over one inhalation [1,2,3]. Under anaesthesia of a suitable depth, apnoea may last 2 to 3 min before carbon dioxide accumulates to stimulate spontaneous ventilation, without causing hypoxaemia due to high fractions of inspired oxygen (FiO_2_). However, the depth must be closely monitored if anaesthesia is maintained by inhalational agents, as patients will not receive these [3].

Anaesthetic induction agents, opioids, or benzodiazepines may be utilised for their respiratory depressant effects in dogs to induce apnoea for thoracic CT [1,4,5,6,7]. Midazolam is an off-license benzodiazepine, which causes various clinical effects depending on the individual and their condition. These may include sedation, anxiolysis, amnesia, muscle relaxation, and seizure control [8]. It may be preferred over other agents due to having a high therapeutic index with minimal cardiovascular side effects [9,10]. The respiratory depression induced by midazolam is also reported to be mild in dogs at doses less than 0.2 mg kg^−1^ and has been better described in the medical literature [7,11,12,13]. This effect may be synergised by concurrent administration of other anaesthetic agents, especially opioids, and so is likely to be more significant under general anaesthesia [12,14]. The speed of administration may be important too, with apnoea reported after rapid intravenous (IV) injection in people [15]. Midazolam’s mechanism of respiratory depression is believed to be agonism of gamma-aminobutyric acid A (GABA_A_) receptors in the medulla, impairing the hypercapnic ventilatory response and relaxing intercostal muscles [11,13,14,16]. In veterinary anaesthesia, midazolam has mainly been investigated as a co-induction or premedication agent [5,6,17,18,19,20,21]. Therefore, its reported respiratory depressant effects have been confounded by those of anaesthetic induction agents; post-induction apnoea (PIA) is a commonly reported side effect of such agents [22]. Despite midazolam having a dose-sparing effect on said induction agents, its co-administration has been associated with an increased incidence of apnoea [5,6,21].

Ventilation techniques can be exploited to induce both apnoea and breath-holding. Commonly, veterinary patients are manually hyperventilated to reduce the hypercapnic ventilatory drive just prior to when apnoea is required, and this may be combined with a breath-hold [2,3,23,24,25]. Another method is to mechanically ventilate patients from the start of anaesthesia to below the apnoeic threshold, the partial pressure of carbon dioxide in arterial blood (PaCO_2_), at which the patient stops ventilating spontaneously. Apnoea may then be achieved by stopping the ventilator. The authors refer to this method as the “interruption of mechanical IPPV” throughout the manuscript. PaCO_2_ typically only needs to be reduced a set amount (4–6 mmHg) from resting levels, which are elevated by the effect of anaesthesia agents [26]. Thus, maintaining normocapnia is usually sufficient but some authors have ventilated to hypocapnia just before inducing a period of apnoea [27,28]. Positive pressure ventilation typically reduces blood pressure by increasing right atrial pressure, and decreasing venous return and consequently stroke volume and cardiac output. This tends to be transitory and insignificant in healthy patients but compromised individuals may have reduced reserves to compensate. Hypotension is more severe during breath-holds, as is pulmonary overdistension, increasing the risk for barotrauma [1,2,29].

In veterinary medicine, apnoea is induced during CT scans to enhance image resolution for detecting pathology by minimising motion blur and organ displacement [1,2,3,28,30]. Image quality is reduced by atelectasis, defined as non-aerated lung predominantly caused by positional compression of pulmonary tissue and the absorption of alveolar gas under anaesthesia, exacerbated by high FiO_2_ [27,31]. Atelectasis can hinder the diagnostic accuracy of CT by masking pathology or being misdiagnosed as pathological increases in image density [1,2].

At the time of writing this manuscript, no veterinary studies could be sourced that evaluated the ability of different methods to achieve apnoea for thoracic CT scans. Furthermore, the description of this application of midazolam in small animals could not be found, despite this being routine at the author’s institution, favoured for its anecdotal efficacy and high therapeutic index. In the absence of a stronger evidence base, methods for apnoea induction are often selected depending on the anaesthetist’s preference. Therefore, the aim of this study was to compare the efficacy of midazolam administration with interruption of mechanical IPPV for inducing apnoea in dogs. The primary hypothesis was that the latter method would induce apnoea of a sufficient duration for the thoracic CT scan in a greater proportion of the study group than midazolam administration. Secondary hypotheses were that interruption of mechanical IPPV would generate a superior image quality due to reduced atelectasis, but cardiovascular side effects would be greater. Specifically, the mean arterial pressure (MAP) would be decreased, compared to following midazolam administration.

## 2. Materials and Methods

### 2.1. Study Design

This was a prospective, controlled, and randomised study. Radiologists were blinded to the intervention group for the induction of apnoea when assessing image quality. Ethical approval was granted by the Central Veterinary Services (CVS) Research Ethical Review Committee (approval number CVS-2023-005). Statutory authority was obtained from the Veterinary Medicines Directorate to conduct a non-commercial clinical trial of midazolam hydrochloride (Hypnovel^®^ 10 mg/2 mL solution for injection, Neon Healthcare Ltd., Hertford, UK, Marketing Authorisation number PL 45043/0037) in dogs (Animal Test Certificate Type S 55944/0002) [15].

### 2.2. Animals

Client-owned dogs undergoing general anaesthesia for thoracic CT were recruited. Written, informed consent was obtained from clients for the enrolment of their dogs in this study and the anonymised storage and utilisation of their associated data for up to 10 years. As per routine clinical veterinary practice, informed consent was also acquired to perform general anaesthesia and the necessary procedures under anaesthesia on subjects, including the administration of off-license medication when indicated, notably midazolam depending on the intervention group randomisation. The inclusion criteria consisted of dogs aged one to 15 years old, weighing between seven and 50 kg, and assigned an American Society of Anesthesiologists (ASA) physical status classification of I to III (with or without emergency, E, status). This incorporated patients considered healthy, and those with mild and severe systemic disease, respectively [32]. Dogs were admitted to a kennel prior to undergoing procedures under general anaesthesia. A physical examination was performed and recent bloodwork interpreted.

### 2.3. General Anaesthesia Protocol

Food was withheld for at least 6 h prior to general anaesthesia; water was provided ad libitum. An IV cannula (Introcan Safety^®^ IV Catheter, B. Braun, Melsungen, Germany) was placed aseptically in advance of the procedure, when tolerated by the patient. Each dog received a tailored anaesthetic plan determined by the responsible anaesthetist, a Diplomate of the European or American College of Veterinary Anaesthesia and Analgesia (ECVAA or ACVAA) or an ECVAA resident working under their supervision. This person was also the allocated observer for the respective patient. Premedication was not standardised; the effect was scored as “none”, “mild”, “moderate”, or “profound”. Following premedication, pre-oxygenation was performed by a facemask if tolerated or flow-by for a minimum of 3 min with 100% oxygen at a uniform flow of 4 L min^−1^. As far as possible, dogs were maintained in sternal recumbency for the duration of anaesthesia to avoid the confounding effect of positional atelectasis.

Propofol (Propofol^®^-Lipuro Vet 10 mg/mL emulsion for injection, B. Braun Melsungen AG, Melsungen, Germany) was then administered as the anaesthesia induction agent as a constant rate infusion (CRI) at 1 mg kg^−1^ min^−1^, whilst continuing oxygen supplementation. The endpoint for the induction of anaesthesia was defined as a reduced jaw tone sufficient for endotracheal intubation, at which point the CRI was stopped. If intubation was attempted but not possible due to inadequate depth, the CRI was resumed until the endpoint was reached. Following intubation, the minimum occlusive volume technique was used to determine the required endotracheal tube (ETT) cuff inflation to form an adequate seal [33]. To achieve this, two breaths were delivered by manual IPPV reaching a maximum PIP of 15 cmH_2_O, as determined by a portable pressure gauge (In-Circuit Pressure Manometer 0–60 cmH_2_O, Burtons Medical Equipment Ltd., Staplehurst, UK) situated on the inspiratory limb of the circle rebreathing system.

**Figure 1:**
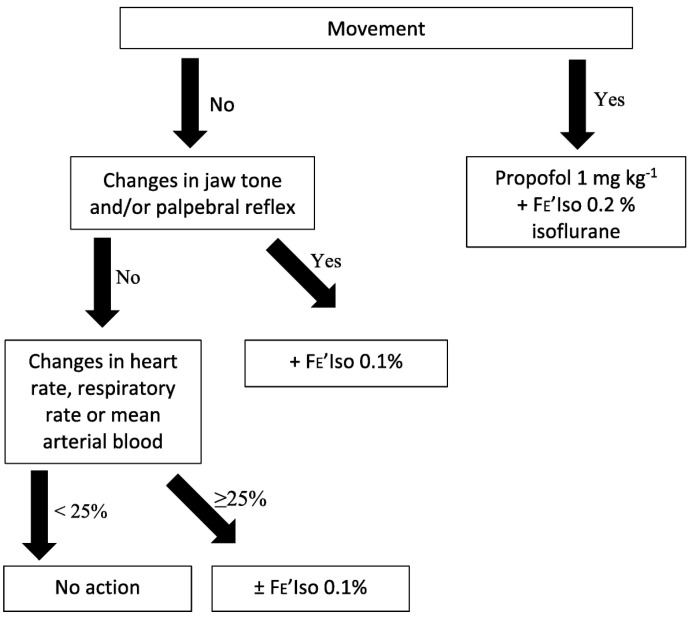
The algorithm to manage the altered depth of dogs under general anaesthesia randomly allocated to either group M or group V, representing different interventions to induce apnoea for thoracic computed tomography, depending on whether signs of lightened depth occur, such as gross movement. Group M, midazolam administration; group V, interruption of mechanical ventilation; FE’Iso, the expired fraction of isoflurane [14].

Anaesthesia was maintained with isoflurane (Iso-vet 1000 mg/mL Inhalation vapour, liquid, Piramal Critical Care Limited, London, UK) in 100% oxygen, delivered by a circle rebreathing system (Darvall, Gladesville, Australia). End tidal isoflurane concentrations (ET_iso_) were monitored and a level of 1% targeted during the stabilisation period prior to the induction of apnoea, as long as this ensured appropriate anaesthetic depth. In the event of a lightened depth, a protocol described by Kropf and Hughes was followed, which has been reproduced below with the original authors’ permission (Figure 1) [14]:

Fresh gas flow was delivered at approximately 100 mL kg^−1^ min^−1^ for the first 15 min, before being reduced to 0.5 L min^−1^ for the remainder of the general anaesthesia. Intravenous fluid therapy (IVFT) (B. Braun Vet Care Hartmann’s Lactated Ringers Solution for infusion for cattle, horses, sheep, goats, pigs, dogs, and cats, B. Braun Melsungen AG, Melsungen, Germany) was administered at a rate of 4 mL kg^−1^ h^−1^ throughout.

As per routine clinical practice, continuous patient monitoring consisted of capnography, pulse oximetry, and electrocardiography, provided by a multi-parameter monitor (Datex-Ohmeda Cardiocap 5 Patient Monitor, Datex-Ohmeda Limited, Leeds, UK). Regular measurements of oscillometric non-invasive blood pressure (NIBP) (Cardell Insight Veterinary Monitor, Midmark, Versailles, OH, USA), rectal temperature, and manual assessments of anaesthetic depth were made concurrently at least every five minutes. Blood pressure cuffs of a width approximating one third the circumference of the dog’s chosen limb or tail were selected.

A cascade of treatment was followed if the intervention point of a MAP less than 65 mmHg lasting 5 min or more was met, in order to avoid significant hypotension, defined as a MAP of less than 60 mmHg [34]:

1. Firstly, a 5 mL kg^−1^ bolus of Hartmann’s solution was administered over 5 min or at a maximum rate of 1200 mL h^−1^. This was repeated if the patient was fluid-responsive but the duration of effect was only transient.
2. Ephedrine 0.1 mg kg^−1^ was administered IV if the patient was not fluid-responsive. This was repeated if it was effective but the duration of effect was only transient.
3. A dopamine CRI was administered at an initial rate of 5 μg kg^−1^ min^−1^ if the patient was refractory to ephedrine. The CRI was increased incrementally to a maximum of 15 μg kg^−1^ min^−1^ if required.
4. Treatment as per the anaesthetist’s discretion was initiated if the patient was refractory to dopamine and the primary investigator was informed.

### 2.4. Study Protocol

Randomisation of blocks of 10 dogs was performed into groups M and V prior to the induction of anaesthesia, representing two different interventions for inducing apnoea for the first thoracic CT scan. In group M, midazolam 0.2 mg kg^−1^ was administered intravenously, followed by 2 mL of sodium chloride 0.9% flush, over a period of 15 s. In group V, mechanical IPPV was initiated immediately following inflation of the ETT cuff using a Penlon Nuffield Series 200 ventilator (Penlon Limited, Abingdon, UK). An inspiratory time of 1 s was fixed but an inspiratory flow and an expiratory time of 2 to 4 s were adjusted to target normocapnia, specifically an ETCO_2_ of 40 mmHg. The inspiratory flow was adjusted so as not to exceed a maximum PIP of 15 cmH_2_O, as determined by the ventilator’s pressure gauge. A standard patient valve was used for dogs weighing 20 kg or more and a Newton valve for patients under 20 kg [35]. Mechanical IPPV was then interrupted to induce apnoea.

If apnoea was not achieved by either method, a “rescue” protocol of rapid intravenous propofol 1 mg kg^−1^ could be administered when requested for image acquisition. These dogs were retained in their original allocation group on an intention-to-treat basis but categorised as having failed with respect to the primary endpoint, the induction of apnoea.

A stopwatch was initiated immediately following each intervention. The onset of apnoea was measured at the point of decrease in ETCO_2_ towards 0 mmHg when it remained there or when the dog visibly stopped breathing. The duration of apnoea was calculated as the difference between the onset of apnoea and the resumption of spontaneous or artificial ventilation or the visualisation of this, at which point the stopwatch was stopped. ETCO_2_ was observed continuously from the capnogram.

Apnoea was induced after a stabilisation period, lasting the duration required to position the patient and perform pre-scans. Simultaneously, baseline cardiorespiratory parameters were recorded and a mean average of three consecutive readings of heart rate, MAP, respiratory rate, ETCO_2_, ET_iso_, saturation of peripheral oxygen (SpO_2_), and PIP in group V was calculated. These recordings were repeated post-intervention.

As a safety measure, intervention thresholds marking significant hypoventilation were defined as follows:

1. Hypoxaemia caused by apnoea, defined as a SpO_2_ of less than 90%, as determined by pulse oximetry. Artificial IPPV was initiated until SpO_2_ reached 95%.
2. An ETCO_2_ of 70 mmHg or more. Artificial IPPV was initiated to target an ETCO_2_ of 60 mmHg or less.
3. Apnoea lasting more than 120 s. Artificial IPPV was initiated.

In group M, if any of these intervention points were met, manual IPPV was applied at an approximate rate of 4 breaths per minute (bpm) and a PIP up to 15 cmH_2_O until the patient ventilated spontaneously and this was adequate. If this intervention did not achieve the target SpO_2_ or ETCO_2_ stated in points 1 and 2 above, mechanical IPPV was initiated in the same manner as described in group V. In group V, mechanical IPPV was simply resumed if an intervention threshold was met.

### 2.5. Radiological Analysis

CT images of the thorax were acquired using a Siemens Somatom 16-slice unit (Siemens Healthcare Limited, Camberley, UK), with a slice thickness of 1.5 mm, a helical mode, pitch 1.2, 130 kVp, an exposure time of 800 ms, and a 512 × 512 matrix, with the scan direction from caudal to cranial, reconstructed with a sharp ‘lung’ algorithm and soft tissue algorithm.

Dogs with CT evidence of significant pulmonary pathology were excluded from radiological analysis. This included single focal soft tissue lesions of a diameter greater than 1 cm, multiple soft tissue nodules, areas of consolidation, thoracic wall, mediastinal or pleural space abnormalities causing lung compression, and atelectasis secondary to bronchial collapse. Scans that did not include the entire lung were also excluded.

**Figure 2:**
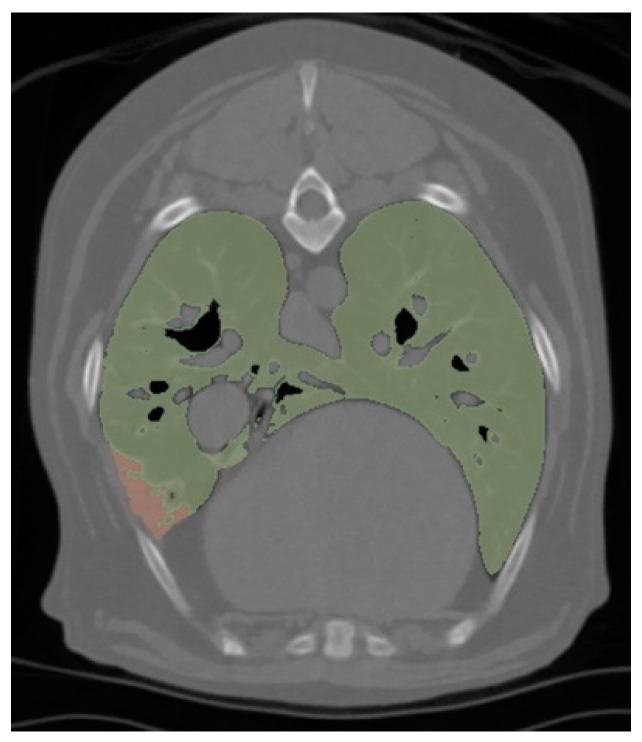
A transverse thoracic computed tomography lung segmentation image obtained from a 3D Slicer analysis software used to measure lung volumes in dogs under general anaesthesia randomly allocated to either group M or group V, representing different interventions to induce apnoea for thoracic computed tomography, demonstrating aerated lung (green) and atelectatic lung (pink). Group M, midazolam administration; group V, interruption of mechanical ventilation.

The aerated lung volume was measured using a semi-automated segmentation and volume measurement tool of an open-source DICOM image analysis software (3D Slicer v5.2.2, www.slicer.org, accessed on 30 August 2024), as demonstrated in Figure 2. Soft tissue reconstruction images were imported into the software and image masks were used to delineate thoracic structures based on Hounsfield unit (HU) values. Thoracic soft tissues and osseous structures were identified at a threshold of −175 to 3071 HU. The trachea and large bronchial airways were identified at a threshold of −1024 to −900 HU. The lungs were then identified at an initial threshold of −900 to −151 HU. This produced an approximate mask of the lungs in the entire study, which was then manually adjusted by one author (MP) to include all areas of pulmonary parenchyma, including hyperattenuating or hypoattenuating lung, whilst excluding tissues identified as the trachea, airway or soft tissue. The final lung mask was then used to calculate the total lung volume and mean CT attenuation of the lungs. Within the total lung volume, a Hounsfield unit threshold of −1024 to −110 HU was used to calculate the volume and mean CT attenuation of aerated lung, excluding areas of atelectasis which were considered greater than −110 HU [31]. Lung aeration was expressed as a percentage of aerated lung in the total lung volume.

A Diplomate of the European College of Veterinary Diagnostic Imaging (ECVDI) and a resident of the same college under their supervision interpreted the thoracic scans. Both were blinded to the intervention group. Images were assessed using a commercial DICOM viewing program (OsiriX, v. 12.5.2. 64bit; PixmeoSARL, CH1233 Bernex, Geneva, Switzerland) in the lung window, predominantly in the acquired transverse plane, with multiplanar reconstructions available as required. Images were assessed for the ‘presence’ or ‘absence’ of respiratory movement artefacts and atelectasis in each lung lobe, agreed on by consensus. Lung lobes were differentiated as the right cranial, right middle, accessory, right caudal, cranial part of the left cranial, caudal part of the left cranial, and left caudal lobes. Atelectasis was defined as a focal area of increased attenuation in the pulmonary parenchyma with an associated reduced lung volume, greater than −110 HU [2,31].

### 2.6. Statistical Analysis

Continuous and ordinal data were assessed for normality graphically and using Shapiro–Wilk tests. The median and interquartile range (IQR) were reported for non-normally distributed data. The mean and standard deviation (SD) were reported for normally distributed data. Categorical data were presented showing the count and percentage. The randomisation of characteristics for the two intervention groups were compared. Differences between the groups for continuous variables were assessed with an unpaired *t* test if normally distributed and a Mann–Whitney test if non-normally distributed. A comparison of categorical variables was made using Chi-square tests and Fisher’s exact tests for variables with less than five observations in a category. A Fisher’s exact test was used to test the primary hypothesis of a difference in the proportion of dogs with a successful induction of apnoea between those in group M and V. If potential confounding factors had been identified that were not accounted for through successful randomisation and were not considered to be on the causal pathway, then multivariable binary logistic regression modelling would have been performed.

The statistical significance was set at probability value *p* < 0.05 throughout. Values were reported to whole numbers, two decimal places or at least one significant figure for values < 0.01. Statistical analyses were performed using GraphPad Prism 10 for macOS, Version 10.2.3 (347), 21 April 2024. The sample size calculation was performed using the open source OpenEpi statistical software, Version 3.01 [36].

## 3. Results

### 3.1. Study Population

A total of 61 dogs were randomised into group M (*n* = 30) and group V (*n* = 31). Descriptive characteristics of this population have been presented in Table 1, indicating that no significant inter-group difference was identified. The most common breeds included crossbreeds (20 dogs), cocker spaniels (7), labradors (5), springer spaniels (3), border collies (3), and Hungarian vizslas (3). Over three quarters of the dogs (47, 77.05%) were assigned an ASA physical status classification of II. Most patients underwent thoracic CT for oncological investigations, to stage a known mass, re-stage a previously excised mass, or screen for suspected neoplasia.

### 3.2. General Anaesthesia Protocol

There was also no significant difference between the anaesthesia protocols of the two groups, as demonstrated in Table 2, except for the administration of midazolam in group M. Premedication agents other than dexmedetomidine hydrochloride (Dexdomitor 0.5 mg/mL Solution for Injection, Orion Corporation, Espoo, Finland) and methadone (Comfortan^®^ 10 mg/mL Solution for Injection for Dogs and Cats, Eurovet Animal Health BV, Bladel, The Netherlands) were utilised in just 11.48% (*n* = 7) of dogs, including in five of the intramuscular (IM) combinations. Ketamine (Anesketin 100 mg/mL solution for injection for dogs, cats and horses, Eurovet Animal Health BV, Bladel, The Netherlands) 1–2 mg kg^−1^ IM, was administered to three dogs, acepromazine (AceSedate 2 mg/mL solution for injection for dogs and cats, Jurox (UK) Limited, Leatherhead, UK) 5–10 µg kg^−1^ IV or IM to two dogs, and butorphanol tartrate (Torbugesic 10 mg/mL Solution for Injection, Zoetis UK Limited, Leatherhead, UK) 0.2–0.3 mg kg^−1^ IV or IM to two dogs in group M in place of methadone. The route of premedication was IV in 86.89% of dogs (*n* = 53); an IM combination was required due to temperament for three dogs in group M and five in group V (*p* = 0.71). A moderate to profound premedication effect was achieved in the majority (95.08%, *n* = 58) of the study population. Only one patient in group M received IV alfaxalone (Alfaxan Multidose 10 mg/mL solution for injection for dogs, cats and pet rabbits, Jurox (UK) Limited, Leatherhead, UK) 1.04 mg kg^−1^ in place of propofol for the induction of anaesthesia; apnoea was successful in this case.

### 3.3. Apnoea

Sample size calculations estimated that a minimum of 60 dogs (30 per group) would be required to gain an 80% power and 95% confidence in detecting a more than five times greater odds of achieving apnoea in group V than group M. This was based on pilot data collected on 14 dogs and other studies in dogs using doses of 0.2–0.4 mg kg^−1^, applying the Kelsey and Fleiss methods [5,6,21,36]. In the pilot group, 50% of the dogs administered midazolam became apnoeic, compared to 85% of those following interruption of mechanical IPPV. The method of apnoea induction in the pilot dogs was not randomised but based on anaesthetist preference.

**Figure 3:**
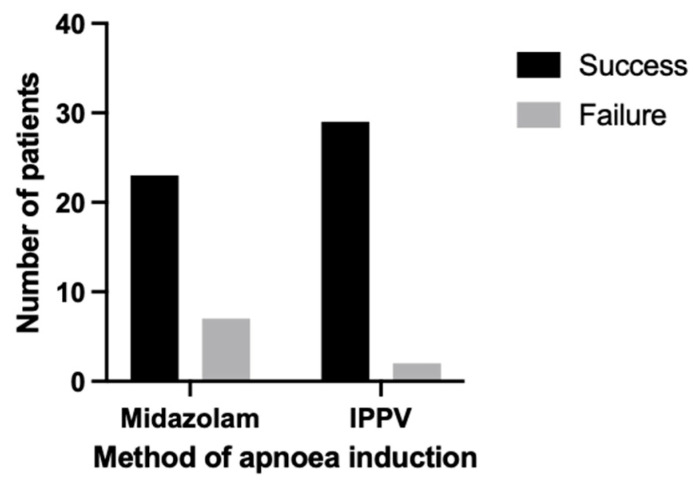
A bar chart demonstrating the success of apnoea during the general anaesthesia of dogs for thoracic computed tomography in terms of the number of dogs in which apnoea was induced (success) versus not induced (failure) against the method of induction of apnoea (total *n* = 61; midazolam *n* = 30; IPPV *n* = 31). Midazolam, administration of midazolam (or dogs randomised to group M). IPPV, intermittent positive pressure ventilation that was interrupted for the CT scan (or dogs randomised to group V).

Apnoea for the thoracic CT scan was achieved in 76.67% of dogs (*n* = 23) in group M, compared with 93.55% (*n* = 29) in group V (*p* = 0.08). Dogs in group V had 4.4 times the odds of achieving apnoea compared to group M (odds ratio = 4.41; 95% confidence interval, CI = 0.93–22.10). These results can be visually compared in Figure 3. The median onset time of midazolam-induced apnoea was 30.00 s (IQR = 20.00–35.00) with a range of 12 to 43 s. Whereas, apnoea was always immediate in group V when it occurred, except in one dog when the onset time was reported to be 13 s (*p* < 0.0001). The duration of apnoea was longer in group V than M with a median duration of 120.00 s (IQR = 86.50–120.00) and 69.00 s (IQR = 40.00–120.00), respectively (*p* < 0.001). The rescue protocol of rapid intravenous propofol 1 mg kg^−1^ was requested by the radiologist in two dogs in group M, where midazolam administration failed to achieve apnoea. Propofol administration induced apnoea in one of these dogs.

### 3.4. Cardiorespiratory Parameters and Complications

Cardiorespiratory parameters differed between group M and group V, as demonstrated by Table 3. Heart rate was significantly higher in group M compared to group V both at baseline (*p* = 0.02) and post-intervention (*p* = 0.0004). Generally, the heart rate increased from baseline to post-apnoea but this was only significant within group M (*p* = 0.01), not group V (*p* = 0.29). In a reciprocal relationship with the heart rate, MAP decreased from baseline to post-intervention by 6.50 mmHg on average in both groups (*p* = 0.83) but this was only significantly different over the study period within group M (*p* = 0.03), not group V (*p* = 0.07). MAP was lower both at baseline (*p* = 0.008) and post-intervention (*p* = 0.002) in group M compared to group V. Overall, there was a low incidence of hypotension. Only one dog experienced hypotension post-intervention in group V. Whereas, three dogs were hypotensive following midazolam administration in group M; one of these was already hypotensive and the other two had a MAP < 65 mmHg at baseline. Interventions to treat the reduced MAP included rapid administration of a 5 mL kg^−1^ crystalloid (Hartmann’s solution) bolus in seven cases, intravenous ephedrine 0.1 mg kg^−1^ in three, and glycopyrrolate 6.5–10 µg kg^−1^ in two.

The respiratory rate was significantly reduced following midazolam administration in group M from a median of 12.00 bpm (IQR = 8.00–15.00) to 7 bpm (IQR = 4.00–10.50) (*p* = 0.001). Consequently, ETCO_2_ was greater in group M both post-intervention and at baseline (*p* < 0.0001). All dogs in group M experienced hypercapnia both pre- and post-intervention for apnoea, except for one case that was hypocapnic (ETCO_2_ 30 mmHg) at baseline and normocapnic post-apnoea. ETCO_2_ was greater than 60 mmHg in 46.67% of dogs (*n* = 14) post-midazolam and the ventilatory intervention threshold of 70 mmHg was met in five patients. Four of these and a further three had a baseline ETCO_2_ exceeding 60 mmHg in group M. None experienced any other significant complications. By contrast, ETCO_2_ never exceeded 60 mmHg in group V but most cases (81%, *n* = 25) experienced mild hypercapnia post-intervention. The percentage increase in ETCO_2_ was greater in group V (23.11%, SD = 8.50) versus M (14.44%, SD = 9.52) (*p* = 0.0004). Desaturation never occurred.

### 3.5. Radiological Analysis

Data were available for 53 cases and are presented in Table 4 below. Eight patients were excluded, four from each group due to pulmonary nodules (*n* = 2), a pulmonary mass (*n* = 1), pneumonia (*n* = 1), a rib lesion causing parenchymal compression (*n* = 1), atelectasis secondary to bronchial collapse (*n* = 2), and incomplete lung capture (*n* = 1). Lower respiratory pathology not detected previously by clinical examination or prior investigation was present in 23.33% (*n* = 7) of group M and 19.35% (*n* = 6) of group V (*p* = 0.70). This included parenchymal lesions or nodules (*n* = 7, including one granuloma), bronchial disease (*n* = 4, including two cases of bronchial collapse, one of which also had bronchomalacia, one case of bronchiectasis with concurrent pneumonia, and one of bronchitis and fibrosis) and bullae (*n* = 2). Some of these cases were not considered significant for exclusion from radiological analysis and incidental osteomata were not considered pathological.

**Figure 4:**
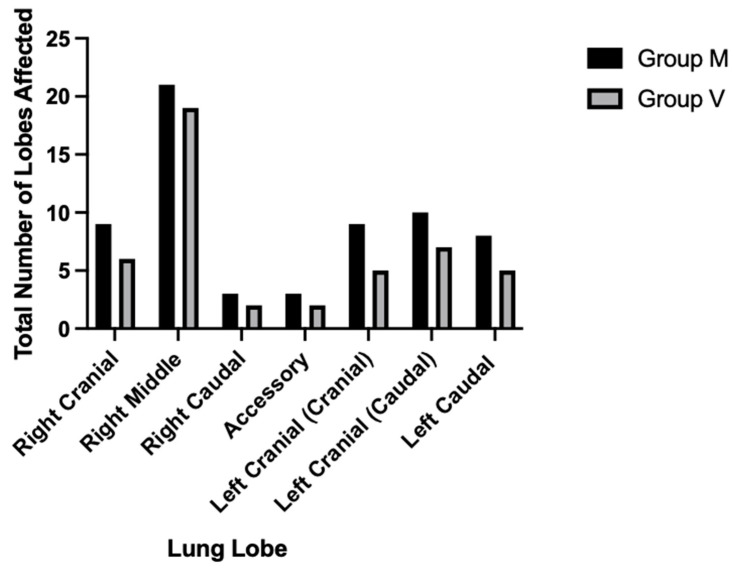
A bar chart of the total number of lobes differentiated anatomically that were affected by atelectasis on the thoracic computed tomography (CT) scans of dogs randomly allocated to either group M or group V, representing different interventions to induce apnoea for thoracic CT under general anaesthesia (total *n* = 53; group M *n* = 26; group V *n* = 27). Group M, midazolam administration; group V, interruption of mechanical ventilation.

Lung aeration was significantly reduced in group M versus group V (*p* = 0.01), with an increased non-aerated lung volume, including when correcting for bodyweight (*p* = 0.03). The distribution of atelectasis was similar between groups (*p* = 0.99), with a predilection for the right middle lung lobe in 70% of dogs (*n* = 19) in group V and 81% (*n* = 21) in group M (*p* < 0.0001), as demonstrated by Figure 4 below. Every dog had an area of atelectasis in at least one lobe, except for two in group M and four in group V that had no atelectasis. Within group M, there was no significant difference in percentage aeration of the lungs of patients experiencing PIA and those that did not (*p* = 0.55) (*n* = 26).

## 4. Discussion

Rapid intravenous midazolam and the interruption of mechanical IPPV instigated from the start of the anaesthetic to maintain normocapnia both achieved apnoea in the majority of dogs, 77% and 94%, respectively. Although approaching statistical significance with a *p* value of 0.08, the primary hypothesis was not accepted at the 5% level. However, interruption of mechanical IPPV had an odds ratio of 4.41 (95% CI 0.93–22.1) for inducing apnoea compared to midazolam administration. Moreover, interruption of mechanical IPPV induces a more controlled period of apnoea due to a predictable, immediate onset and longer, titratable duration, reduced cardiorespiratory side effects and improved image quality. Mechanical IPPV can also be resumed between CT scans and stopped again when a subsequent period of apnoea is required.

The difference in success of the two methods compared to induce apnoea for thoracic CT was arguably not clinically significant either. Indeed, a post-hoc sample size calculation determined that at least a total of 140 dogs (70 dogs per group) would have been required to detect a significant inter-group difference. A type II error may have feasibly occurred, as in this study midazolam outperformed previously reported success rates utilised for the initial sample size calculation. This analysis was based on previously determined success rates of midazolam of 50% or less [5,6,21].

The duration of apnoea was significantly longer when instigated by the interruption of mechanical IPPV than by the administration of midazolam (*p* < 0.001). As the apnoeic threshold is increased under general anaesthesia, targeting a higher ETCO_2_ during mechanical IPPV may have been sufficient to achieve a similar success rate of apnoea induction [26]. Another advantage of interruption of mechanical IPPV is that the anaesthetist can set a shorter duration of apnoea, resuming mechanical IPPV as soon as the CT scan is complete. By contrast, the duration of midazolam-induced apnoea was more variable but shorter on average, possibly due to the increased ETCO_2_ and hence spontaneous respiratory drive pre-intervention [26]. The onset of apnoea following the interruption of mechanical IPPV was also immediate, except for one dog where the onset was recorded as 13 s. Whereas, the CT scan had to be delayed until the onset of midazolam-induced apnoea in group M, which varied but averaged at 30 s (*p* < 0.0001). The lag on the capnogram may have resulted in an overestimation of the onset time for midazolam-induced apnoea, as a stopwatch was commenced immediately after administration.

Generally, midazolam caused hypoventilation, even when complete apnoea was not achieved. Indeed, there was a significant reduction in respiratory rate following midazolam administration (*p* = 0.001). In line with decreasing respiratory rates, midazolam resulted in a greater ETCO_2_ post-intervention but also at baseline (*p* < 0.0001). ETCO_2_ exceeded 60 mmHg in nearly half of the dogs post-midazolam but this tended to be short-lived. Valid inter-group comparisons cannot be made here, as ventilation was controlled in group V to achieve normocapnia; consequently, ETCO_2_ never exceeded 60 mmHg even post-apnoea. Whereas, midazolam-induced apnoea was only implemented in spontaneously breathing patients and hypercapnia is more likely with this mode of ventilation under general anaesthesia [26]. This may have contributed to midazolam being less likely to induce apnoea, as the spontaneous respiratory drive was greater [26]. A change in ETCO_2_ should be anticipated with any period of apnoea and the percentage increase was actually greater in group V, when comparing initial post-intervention values (*p* = 0.0004), likely due to the increased duration of apnoea. Despite this, the degree of hypercapnia in group V was less and it is an advantage of the technique that ETCO_2_ levels may be controlled more closely.

The secondary hypothesis that the interruption of mechanical IPPV would cause more cardiovascular disturbance than midazolam was disproven. No significant changes in cardiovascular variables were identified over the course of the study period in group V. However, initial measurements did not truly represent a “baseline” in this group, as mechanical IPPV was commenced immediately post-induction of anaesthesia. Similarly, post-apnoea recordings were often taken when IPPV had already been resumed due to persistent apnoea. Accounting for differences at baseline, the percentage increase in heart rate was still significantly greater after midazolam but the decrease in MAP was not, when compared to group V. Midazolam-associated positive chronotropy may have occurred secondary to hypercapnic sympathetic stimulation or to compensate for the decrease in MAP post-intervention in group M (*p* = 0.03). The clinical relevance of these findings is debatable as increases in heart rate were mild, never exceeding 125 beats per minute, and there were only three cases of hypotension following midazolam administration. Notably, these changes may be more significant in a higher risk study population, especially when synergised with other anaesthetic agents. For instance, midazolam has been associated with severe hypotension in neonates and critically ill people, especially when administered rapidly or with fentanyl [37].

Midazolam has previously been demonstrated to increase heart rate, specifically by 15% following IV doses of 0.25, 1, and 10 mg kg^−1^ [9]. A reduction in blood pressure is generally only observed at supraclinical doses (≥1 mg kg^−1^), although Glisson detected an 8–10% decrease in MAP after 0.2 mg kg^−^^1^ [38]. Midazolam blunts the sympathoadrenal response to hypotension but cardiac output tends to be maintained or even increased by a compensatory tachycardia and mobilisation of the splanchnic circulation [9,38,39]. Other authors have also identified a trend of increasing heart rate and decreasing blood pressure following midazolam co-induction, despite a reduction in the anaesthetic induction agent dose requirement [5,21]. Suggested mechanisms for this include a reduced systemic vascular resistance and negative inotropy, exacerbated under conditions of hypovolaemia [5,9,20,21,40]. Furthermore, the initial ET_iso_ of 1% may have been superfluous following the midazolam administration, considering its demonstrated minimum alveolar concentration (MAC)-sparing effect, and so patients may have been relatively more vasodilated [14,21].

A further advantage of the interruption of mechanical IPPV over midazolam administration was that pulmonary aeration was greater. Consequently, it may reduce the likelihood of missing pathology obscured by atelectatic or non-aerated areas. Having said this, non-aerated lung occupied just a small fraction of the total volume in both groups, so the clinical value of this significant difference is debatable. The right middle lung lobe was most commonly affected by atelectasis in both groups, supporting reports of it being the most prone to collapse in the literature [41]. The volume of non-aerated lung due to absorption atelectasis could have been further reduced in this study by using an oxygen–air mixture instead of 100% oxygen to decrease the FiO_2_ [27,29]. Thoracic imaging under sedation has also been demonstrated to cause less atelectasis than that typically developing within eight minutes of general anaesthesia [42,43].

Midazolam-induced bradypnoea may be sufficient to eliminate respiration blur on CT even when apnoea is not achieved, as demonstrated by three dogs in group M. Drug-induced hypoventilation has been reported to prevent respiration blur in dogs sedated with dexmedetomidine and butorphanol, when scanned at high speed with a multi-detector helical CT machine [42]. Overall, there was no significant difference in the incidence of respiratory motion artefact between groups, which correlated with the failure of apnoea. However, the presence of respiration blur despite successful apnoea was recorded in one patient per group. This may be explained by apnoea of a duration too short for the thoracic CT sequence or the patient having taken very shallow breaths not detected by capnography.

Overall, randomisation of the subject population was performed successfully to account for a priori considered confounding factors. No significant difference was detected between groups M and V, except for factors considered to be on the causal pathway and the general anaesthetic protocols of both groups were near identical. However, the study was limited by the non-standardisation of the premedication. Where possible, tailored agents and doses were titrated to achieve at least a moderate effect to account for individual-dependent responses but assessment of this was subjective. It could have been improved by use of a validated sedation score, such as the composite one adapted by Grint et. al. and later validated by Wagner et al. [44,45]. Notably, the use of additional premedication agents, namely acepromazine and ketamine, was associated with apnoea in every case but this observation was not statistically significant (*p* = 0.58). Whilst the respiratory depression caused by these drugs individually is reportedly minimal, potentiation may occur in combination with other sedatives [46]. The success of apnoea in these cases may also be explained by the increased dosages administered intramuscularly.

Limitations of the study include that the primary observer was not blinded to the intervention group for practical and safety reasons. In addition, there was a total of four observers but all were ECVAA residents or ECVAA/ACVAA Diplomates and most cases (*n* = 52) were collected by the primary investigator, an ECVAA resident. Furthermore, objective outcome measures were clearly defined to minimise any potential observer bias and inter-observer variability. PIP was measured crudely from the ventilator’s pressure gauge and spirometry could have been utilised to improve accuracy. However, this probably did not affect primary outcome measures, as patients were simply ventilated to reach the apnoeic threshold. An accurate measurement of PIP is more important when considering its effect on CT image quality, as lung aeration may be reduced at lower values [1].

## 5. Conclusions

The success of apnoea induction by midazolam administration and the interruption of mechanical IPPV at normocapnia were not significantly different, at the 5% significance level. However, interruption of mechanical IPPV indicated a tendency towards increased odds of apnoea induction compared to midazolam administration. Benefits of interruption of mechanical IPPV over midazolam included a longer duration and a quicker (immediate) onset of apnoea, improved cardiovascular stability, and increased pulmonary aeration. IPPV could also be resumed and stopped again as required. Moreover, a stricter control of ETCO_2_ levels prevented the greater degree of hypercapnia observed following midazolam administration. The incidence of adverse effects was low in both groups, although midazolam increased heart rate and decreased MAP post-administration unexpectedly more than IPPV. Whilst generally considered to have minimal cardiorespiratory side effects, midazolam may therefore exacerbate existing cardiovascular instability, especially when synergised with other anaesthetic agents. Any period of apnoea and methods of induction are not devoid of potential complications, so a risk–benefit analysis should be performed beforehand.

## Figures and Tables

**Table 1:** Descriptive characteristics in dogs randomly allocated to either group M or group V representing different interventions to induce apnoea during general anaesthesia for thoracic computed tomography (total *n* = 61; group M *n* = 30; group V *n* = 31). Group M, midazolam administration; group V, interruption of mechanical ventilation; *p* value, probability value; ASA, American Society of Anesthesiologists physical status classification; SD, standard deviation; IQR, interquartile range.

		Group M	Group V	*p* Value
**Age (years)**		8.80 (SD = 3.09)	8.73 (SD = 3.04)	0.94
**Sex (%):**	**Male**	60.00	54.84	0.68
	**Female**	40.00	45.16	
**Breed:**	**Crossbreed**	9	11	
	**Cocker Spaniel**	5	5	0.90
	**Other**	16	15	
**Body Condition Score (/9)**		5 (IQR 4–7)	5 (IQR = 4–6)	0.94
**Bodyweight (kg)**		22.48 (SD = 10.34)	21.62 (SD = 8.86)	0.73
**ASA I**		0	1	
**ASA II**		24	23	>0.$\dot{9}$
**ASA III**		6	7	
**Reason for CT:**	**Oncological**	65.63	62.50	0.80
	**Other**	34.37	37.50	
**Concurrent Medication (%)**		43.33	58.06	0.25

**Table 2:** A comparison of components of the general anaesthesia protocol in dogs randomly allocated to either group M or group V, representing different interventions to induce apnoea during general anaesthesia for thoracic computed tomography (total *n* = 61; group M *n* = 30; group V *n* = 31). Group M, midazolam administration; group V, interruption of mechanical ventilation; *p* value, probability value; IQR, interquartile range; induction, the induction of general anaesthesia; ET_iso_, end tidal isoflurane concentration.

	Group M	Group V	*p* Value
**Dexmedetomidine Dose (µg kg^−1^)**	2 (IQR = 2–2.25)	2 (IQR = 2–2)	0.85
**Methadone Dose (mg kg^−1^)**	0.2 (IQR = 0.2–0.2)	0.2 (IQR = 0.2–0.2)	>0.$\dot{9}$
**Other Premedication Agents (%)**	13.33	9.68	0.71
**Propofol Dose (mg kg^−1^)**	2.00 (IQR = 1.50–2.53)	2.00 (IQR = 1.60–2.12)	0.997
**Premedication to Induction (min)**	11.50 (IQR = 7.75–13.00)	9.00 (IQR = 7.00–12.25)	0.30
**Induction to Apnoea (min)**	21.00 (IQR = 17.00–27.00)	17.00 (IQR 16.00–24.50)	0.10
**ETiso Baseline (%)**	1.00 (IQR = 0.97–1.03)	1.00 (IQR = 0.98–1.03)	0.63
**ETiso Post-Intervention (%)**	0.99 (IQR = 0.92–1.02)	0.96 (IQR = 0.94–1.00)	0.33

**Table 3:** Cardiorespiratory parameters pre- (baseline) and post-intervention in dogs randomly allocated to either group M or group V, representing different interventions to induce apnoea for thoracic computed tomography under general anaesthesia (total *n* = 61; group M *n* = 30; group V *n* = 31). Group M, midazolam administration; group V, interruption of mechanical ventilation; *p* value, probability value; IQR, interquartile range; SD, standard deviation; bpm, beats or breaths per minute when referring to heart rate or respiratory rate, respectively; MAP, mean arterial pressure; ETCO_2_, end-tidal carbon dioxide concentration; SpO_2_, saturation of peripheral oxygen.

	Group M	Group V	*p* Value
**Apnoea success (%)**	93.55	76.67	0.08
**Apnoea onset (s)**	30.00 (IQR = 20.00–35.00)	0.00 (IQR = 0.00–0.00)	<0.0001
**Apnoea duration (s)**	69.00 (IQR = 40.00–120.00)	120.00 (IQR = 86.50–120.00)	<0.001
**Heart rate baseline (bpm)**	74.00 (IQR = 64.50–84.00)	59.50 (IQR = 52.50–72.25)	0.02
**Heart rate post-intervention (bpm)**	89.50 (IQR = 71.75–100.00)	68.00 (IQR = 51.50–78.50)	0.0004
**Heart rate absolute change (bpm)**	12.63 (SD = 9.87)	5.72 (SD = 6.60)	0.002
**Heart rate percentage change (%)**	+19.16 (SD = 16.68)	+9.62 (SD = 11.04)	0.01
**MAP baseline (mmHg)**	82.65	91.87	0.008
**MAP post-intervention (mmHg)**	75.87	85.69	0.002
**MAP absolute change (mmHg)**	−6.50 (IQR = −9.25–−2.75)	−6.50 (IQR = −10.00–−3.00)	0.83
**MAP percentage change (%)**	−7.30 (IQR = −11.66–−3.13)	−7.62 (IQR = −9.79–−3.81)	0.81
**Respiratory rate baseline (bpm)**	12.00 (IQR = 8.00–15.00)	13.00 (IQR = 12.00–16.00)	0.01
**Respiratory rate post-intervention (bpm)**	7.00 (IQR = 4.00–10.50)	13.00 (IQR = 12.00–17.00)	<0.0001
**ETCO_2_ baseline (mmHg)**	52.50 (IQR = 49.88–60.25)	40.00 (IQR = 39.00–40.00)	<0.0001
**ETCO_2_ post-intervention (mmHg)**	59.00 (IQR = 57.00–63.25)	45.50 (IQR = 45.00–47.25)	<0.0001
**ETCO_2_ absolute change (mmHg)**	7.00 (IQR = 3.50–11.00)	6.00 (IQR = 4.88–8.25)	0.43
**ETCO_2_ percentage change (%)**	12.61 (IQR = 6.66–21.68)	15.00 (IQR = 12.46–20.23)	0.10
**SpO_2_ baseline (%)**	97.00 (IQR = 96.00–99.00)	98.00 (IQR = 96.00–98.00)	0.71
**SpO_2_ post-intervention (%)**	97.00 (IQR = 96.00–98.25)	97.00 (IQR = 96.00–99.00)	0.66

**Table 4:** The radiological analysis of the pre-contrast thoracic computed tomography (CT) scans of dogs randomly allocated to either group M or group V, representing different interventions to induce apnoea for thoracic CT under general anaesthesia (total *n* = 53; group M *n* = 26; group V *n* = 27), including lung volume measurements made using the 3D Slicer image analysis software. Group M, midazolam administration; group V, interruption of mechanical ventilation; *p* value, probability value; IQR, interquartile range; SD, standard deviation; HU, Hounsfield Unit; remaining abbreviations are explained in the table.

	Group M	Group V	*p* Value
**Respiratory Motion (% of cases)**	19.23	7.41	0.25
**Total Lung Volume (TLV) (cm^3^)**	1012 (IQR = 698.4–1553)	951.4 (IQR = 566.4–1274)	0.51
**TLV/Body Weight (cm^3^ kg^−1^)**	48.44 (IQR = 35.92–61.76)	48.04 (IQR = 36.83–60.68)	0.91
**Aerated Lung Volume (ALV) (cm^3^)**	1100 (SD = 116.5)	1018 (SD = 106.9)	0.61
**ALV/Body Weight (cm^3^ kg^−1^)**	48.08 (IQR = 35.85–61.61)	47.95 (IQR = 36.82–60.66)	0.88
**Non-Aerated Lung Volume (NLV) (cm^3^)**	3.66 (IQR = 1.11–5.47)	1.49 (IQR = 0.39–2.60)	0.02
**NLV/Body Weight (cm^3^ kg^−1^)**	0.14 (IQR = 0.09–0.22)	0.10 (IQR = 0.02–0.13)	0.03
**Aeration (%)**	99.68 (IQR = 99.53–99.79)	99.82 (IQR = 99.68–99.96)	0.01
**Attenuation of Total Lung Volume (HU)**	−724.2 (IQR = −750.0–−679.1)	−716.1 (IQR = −760.7–−676.9)	0.84
**Attenuation of Aerated Lung Volume (HU)**	−724.9 (IQR = −751.8–−679.8)	−714.3 (IQR = −760.8–−677.2)	0.87
**Number of Lobes with Atelectasis**	2.00 (IQR = 1.00–3.25)	1.00 (IQR = 1.00–2.00)	0.16

## Data Availability

The data presented in this study are available on request from the corresponding author for up to 10 years due to restrictions on the maximum time period consented for data retention.
